# An electrophysiological biomarker for the classification of cataract-reversal patients: A case-control study

**DOI:** 10.1016/j.eclinm.2020.100559

**Published:** 2020-10-06

**Authors:** Suddha Sourav, Davide Bottari, Idris Shareef, Ramesh Kekunnaya, Brigitte Röder

**Affiliations:** a Biological Psychology and Neuropsychology, University of Hamburg, Hamburg, Germany; b IMT School for Advanced Studies Lucca, Lucca, Italy; c Jasti V Ramanamma Children's Eye Care Center, Child Sight Institute, L V Prasad Eye Institute, Hyderabad, India

**Keywords:** Congenital cataract, Cataract, Sight recovery, Biomarker, Visual deprivation, Extrastriate processing, Pediatric cataract

## Abstract

**Background:**

Untreated congenital blindness through cataracts leads to lasting visual brain system changes, including substantial alterations of extrastriate visual areas. Consequently, late-treated individuals (> 5 months of age) with dense congenital bilateral cataracts (CC) exhibit poorer visual function recovery compared to individuals with bilateral developmental cataracts (DC). Reliable methods to differentiate between patients with congenital and developmental cataracts are often lacking, impeding efficient rehabilitation management and introducing confounds in clinical and basic research on recovery prognosis and optimal timing of surgery. A persistent reduction of the P1 wave of visual event-related potentials (VERPs), associated with extrastriate visual cortical activity, has been reported in CC but not in DC individuals. Using two experiments, this study developed and validated P1-based biomarkers for diagnosing a history of congenital blindness in cataract-reversal individuals.

**Methods:**

Congenital and developmental cataract-reversal individuals as well as typically sighted matched controls took part in a first experiment used for exploring an electrophysiological biomarker (*N*_CC_ = 13, *N*_DC_ = 13, *N*_Control_ = 26). Circular stimuli containing gratings were presented in one of the visual field quadrants while visual event-related potentials (VERPs) were recorded. Two biomarkers were derived from the P1 wave of the VERP: (1) The mean of the normalized P1 amplitude at posterior electrodes, and (2) a classifier obtained from a linear support vector machine (SVM). A second experiment with partially new CC/DC individuals and their matched controls (*N*_CC_ = 14, *N*_DC_ = 15, *N*_Control_ = 29) was consecutively used to validate the classification based on both biomarkers. Performance of the classifiers were evaluated using receiver operating characteristic (ROC) curve analyses. All cataract-reversal individuals were tested after at least one year of vision recovery.

**Findings:**

The normalized P1 amplitude over posterior electrodes allowed a successful classification of the CC from the DC individuals and typically sighted controls (area under ROC curve, AUC = 0.803 and 0.929 for the normalized P1 amplitude and the SVM-based biomarker, respectively). The validation for both biomarkers in experiment 2 again resulted in a high classification success (AUC = 0.800 and 0.883, respectively for the normalized P1 amplitude and the SVM-based biomarker). In the most conservative scenario involving classification of CC from DC individuals in a group of only cataract-reversal individuals, excluding typically sighted controls, the SVM-based biomarker was found to be superior to the mean P1 amplitude based biomarker (AUC = 0.852 compared to 0.757 for the mean P1 based biomarker in validation). Minimum specificity obtained was 80% across all biomarkers.

**Interpretation:**

A persistent reduction of the P1 wave provides a highly specific method for classifying cataract patients post-surgically as having suffered from bilateral congenital vs. bilateral developmental cataracts. We suggest that using the P1 based non-invasive electrophysiological biomarker will augment existing clinical classification criteria for individuals with a history of bilateral congenital cataracts, aiding clinical and basic research, recovery prognosis, and rehabilitation efforts.

**Funding:**

German Research Foundation (DFG) and the European Research Council (ERC).

**Research in context**

**Evidence before this study**

We searched PubMed on January 25, 2020, for articles published in English, French, German, Hindi, Portuguese or Spanish, without publication time constraints, using the terms ("child" AND "cataract" AND ("diagnosis" OR "outcome") AND ("VEP" OR "EEG" OR "VERP" OR "Evoked Potential" OR "Event-Related Potential")). One study involving visual event-related potentials (VERPs) reported more severe visual deficits in children after a history of congenital cataracts than for congenital squint, but did not report any relationship between the electrophysiological and clinical findings. Despite some studies applying preoperative VERP measures to predict postoperative visual acuities, all in adults, we found none that used an age-independent electrophysiological measure to differentiate congenital and developmental etiologies in pediatric cataract populations.

**Added value of this study**

We report that a persistent reduction of the *P1 wave* in VERPs can be used to develop age-independent electrophysiological biomarkers for diagnosing a congenital vs. developmental etiology of visual deprivation. To the best of our knowledge, this is the first time that reported persistent changes in extrastriate cortical processing has been used to derive non-invasive, robust, and cost-sensitive biomarkers after sight restoration for a history of congenital bilateral dense cataracts and congenital pattern vision deprivation.

**Implications of all the available evidence**

An equal period of visual deprivation leads to substantially graver and potentially permanent consequences for visual recovery if it is congenital compared to when the deprivation is developmental. Qualitatively different and more intense rehabilitation approaches are likely required for individuals with a history of congenital bilateral visual deprivation compared to those with a developmental deprivation. Without differentiation between these two distinct types of visual deprivation, as frequently observed in extant studies, both clinical and basic research bear the risk of producing inconsistent and uninterpretable results. Electrophysiological methods as developed in the present study could be a helpful tool for obtaining an independent data point for determining the history of visual deprivation, aiding recovery prognosis and rehabilitation effort planning as well as clinical and basic research in pediatric cataract populations.

Alt-text: Unlabelled box

## 1 Introduction

Cataract is one of the leading causes of treatable childhood blindness, with children from low-income countries bearing a disproportionate disease burden [1,2]. As the developing visual system is especially susceptible to aberrant visual information [3], a transient period of blindness which might have minimal effects in adults can indelibly alter visual brain systems in children. Childhood cataracts are therefore rigorously treated, since each week of additional visual deprivation in the first 3–4 months of life markedly and persistently reduces postsurgical visual acuity while increasing the probability of developing strabismus and nystagmus [4,5]. Moreover, individuals treated for congenital bilateral dense cataracts (CC), especially those treated late (defined here as treated after the age of 5 months), fare substantially worse than individuals treated for bilateral developmental cataracts (DC), exhibiting not only persistently reduced visual acuity after surgery [6,7], but additionally enduring significant deficits in higher-level visual processing due to altered neural circuits [8], [9], [10], [11]. DC individuals have been reported to attain higher visual capabilities, partially close to the level of typically sighted controls especially when transient visual deprivation occurs sufficiently late so as to avoid *sensitive periods* in visual development [9,12,13].

Despite the consensus for early intervention, children in developing countries are frequently diagnosed and operated later due to infrastructural and socioeconomic factors. In India, the mean ages at surgery for congenital and developmental cataracts have been reported to be about 48 and 99 months respectively, with only 20% of the CC children undergoing surgery within the first year of life [14]. Later treatment not only lowers the ceiling of visual recovery, particularly in CC individuals, but additionally increases the potential of incorrect diagnosis of cataract onset e.g. due to symptom overlap at presentation and in some cases, cataract absorption [15]. Expedited intervention for cataracts causing significant vision reduction is urged for later-diagnosed individuals regardless of congenital or developmental etiology, but prognosis of postsurgical vision recovery strongly depends on whether cataracts existed at birth or emerged later. On the one hand, the visual system seems to have a remarkable ability to recover basic visual functions even after long periods of congenital blindness. For example, color discrimination and key aspects of retinotopic processing in early visual areas have been observed to recover to a relatively high level after congenital cataract surgery [8,12,16]. On the other hand, persistent deficits in higher-level visual processes, such as face and global motion processing, have been reported after sight restoration following a history of congenital cataracts [9,17]. Late-treated CC individuals furthermore require additional and more intensive rehabilitation efforts, an issue that has to date often been neglected.

Categorizing congenital from developmental vision deprivation, such as through cataracts, is of crucial importance for not only prognosis, rehabilitation, and caregiving-related issues: A reliable classification of late-treated cataract individuals additionally aids clinical and basic research alike. Clinical studies investigate surgery or treatment procedures for achieving optimal outcomes and examine factors that should receive consideration in multicenter studies to obtain comparable results [18], [19], [20]. Since surgery outcomes depend on whether individuals with a history of congenital or developmental cataracts were included, and since the relative number varies across centers, the lack of well-stratified groups impedes reliable and valid conclusions about treatment procedures. Thus, a highly specific classification using an identical electrophysiological biomarker across patients would considerably improve clinical practice and research. The availability of an electrophysiological biomarker would augment initial clinical diagnosis procedures based on anamnestic information from patients, parents and primary healthcare providers, if available, as well as criteria such as the presence of nystagmus and type of strabismus present [21,22].

Moreover, for basic research on the neural mechanisms of sensitive periods in visual development, a reliable distinction between congenital and developmental cataract patients is essential. The increasing availability of noninvasive neuroscientific methods now allows investigating neural circuit structures and functions and their experience dependent development in humans [23]. However, unlike in non-human animal research, where experience is systematically manipulated, research involving humans must make use of natural variations of visual experience during development. Thus, it is of utmost importance to identify and investigate homogenous groups of patients with regard to the onset and degree of visual deprivation. For example, impairment of a visual or neural function after congenital but not developmental visual deprivation provides strong evidence of an early sensitive period for the development of that function, after which acquiring the skill and related brain functions becomes difficult or is incomplete.

Due to incompleteness of patient information records and the inherent difficulty of pinpointing an age of onset in individuals whose cataracts were only treated months and years after onset, existing research has often lumped congenital and developmental etiologies together under terms like *childhood cataract* or *congenital/developmental cataract* [14], increasing the probability of confounding via averaging effects across two distinct groups and thus deriving invalid conclusions in both clinical and basic research. However, despite an established history of using visual event-related potentials (VERPs) for research and clinical use, an electrophysiological method for classifying congenital from developmental visual deprivation is yet lacking.

We have recently demonstrated that the first cortical event-related potential indexing retinotopic processing, the *C1 wave,* is present in CC individuals and is elicited with a latency which was indistinguishable between CC individuals and typically sighted controls. Despite some alteration in scalp voltage distribution, the results suggested a spared basic retinotopic organization of early visual cortex (and associated visual processing) even after long periods of congenital pattern vision blindness [12]. By contrast, we found a strong reduction of the group mean amplitude of the second, positive-going P1 wave of the VERP in CC but not in DC individuals compared to normally sighted controls [9,12,24]. The P1 wave has been hypothesized to be generated in extrastriate visual cortex [25], which non-human primate work has suggested to be particularly sensitive to early visual deprivation [26].

In two separate experiments involving simple high contrast grating stimuli, the present study investigated whether the P1 amplitude can serve as an electrophysiological biomarker for distinguishing individuals with a history of a transient congenital vs. developmental phase of cataracts. In both EEG experiments, individuals operated for either congenital or developmental bilateral cataracts, as well as typically sighted controls matched for age, sex, and handedness, saw visual stimuli presented in a random order to one of the visual field quadrants while they were engaged in a detection task. We hypothesized that individuals treated for congenital cataracts can be distinguished from the individuals treated for developmental cataracts and typically sighted controls based on the P1 amplitude, both with a high sensitivity and specificity.

## 2 Methods

### 2.1 Participants

Experiments 1 and 2 developed and validated the electrophysiological biomarkers, respectively. We aimed for 15 participants per group based on a priori power analyses involving additional stimuli not analyzed here. In experiment 1, 13 individuals with a history of dense, total, and bilateral congenital cataracts and subsequent sight restoration took part (CC; mean age = 17.85 years, range = 8 – 37 years, 2 female, 1 left-handed). The process for classifying a participant as CC started if bilateral dense cataracts at presentation were observed including fundus invisibility, or if the participant had partially absorbed cataracts, accompanied by nystagmus. Crucially, anamnestic information from family members and primary healthcare providers, including family history and behavioral history of the participant as a child, was used as corroborating evidence. The existence of strabismus, such as esotropia, additionally helped us classify the CC individuals, in combination with all the other information [27]. Participants were only included when a very high confidence in the clinical diagnosis existed, and excluded otherwise. The mean duration of blindness (= age of surgery) in the CC individuals was 38 months, ranging from 1 month to 17.75 years, and the mean time since surgery was 15.08 years (range: 2.08 – 35.67 years). The mean geometric visual acuity in the CC group was 0.251 (decimal, range: 0.051 – 0.7). A second group of 16 participants who had bilateral developmental cataracts and had undergone cataract-removal surgery took part in the study (DC group). The data of three of these participants were rejected because of incomplete data acquisition (one had a history of seizure and developmental delay, one wanted to abort the experiment shortly after the start and the third participant was not able to look straight ahead). The remaining 13 participants had a mean age of 16 years (range = 11 – 24 years, 5 female, all right-handed). The age of the DC individuals did not significantly differ from the CC individuals, two-tailed *t-*test, *t*(16.074) = 0.656, *p* = .521. The geometric mean visual acuity in the DC group was 0.739 (decimal, range = 0.44 – 1.00), and this group had a significantly better visual acuity than the CC group (one-tailed *t-*test after converting the acuities to LogMAR units, *t*(15.661) = 5.130, *p* < .001). The mean age at surgery in the DC group was 9.78 years (range = 2 – 17.33 years) and the mean time since surgery was 6.63 years (range = 1.42 – 22 years). Additionally, a group of 26 control participants matched for age, sex, and handedness with a CC or DC participant took part in the experiment (mean age = 17.04 years, range = 7 – 34 years, 7 female, 1 left-handed). All controls had normal or corrected-to-normal vision and were free from sensory impairments. All subjects were free of neurological problems as assessed by reports of the participants or their legal guardians and a general clinical assessment in case of the cataract reversal individuals. Although we matched the control participants to individual participants from the CC or the DC group, for classification purposes this group served as a homogenous participant group.

**Fig. 1:**
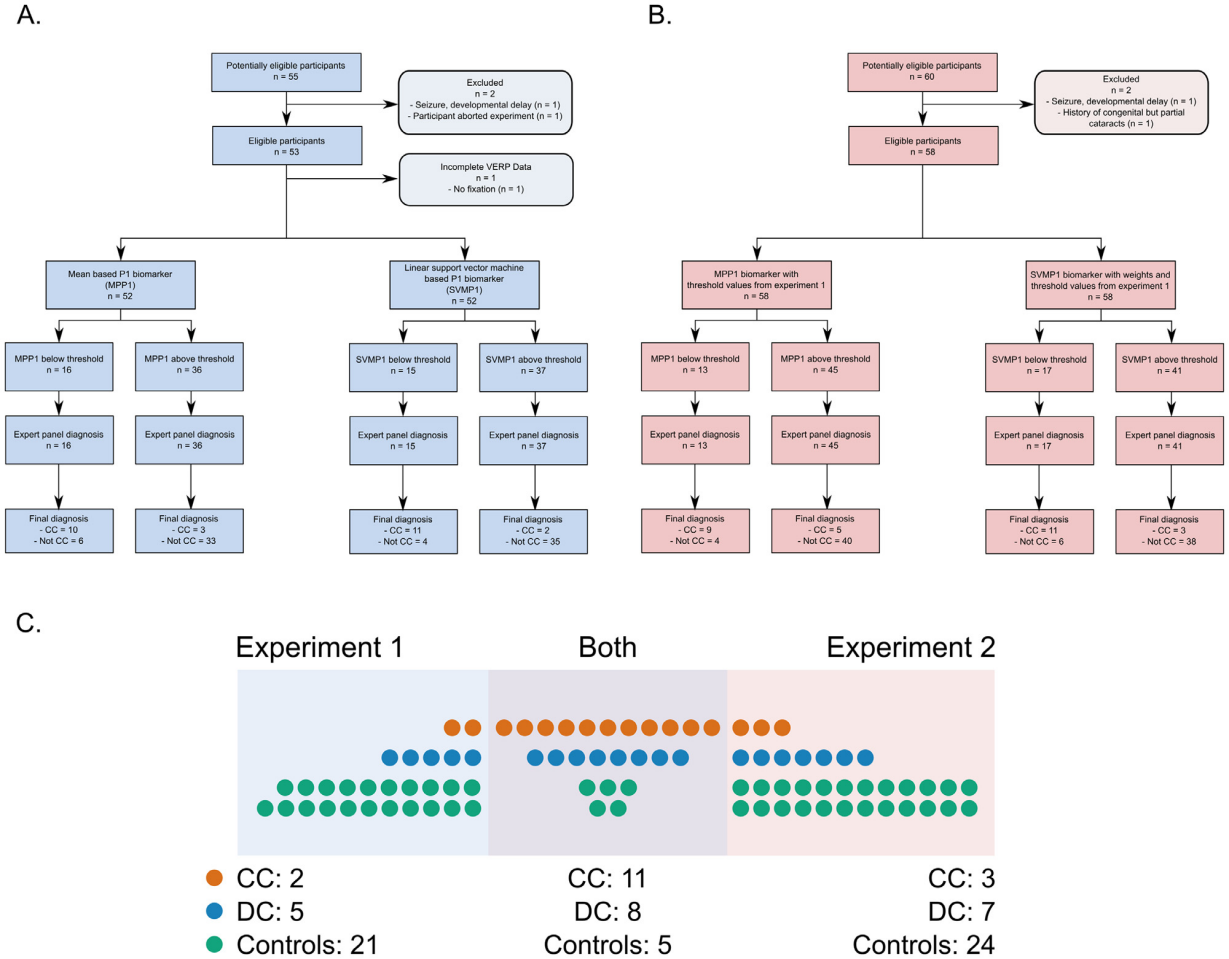
**A.** Standards for Reporting of Diagnostic Accuracy (STARD) flow diagram for P1 amplitude based diagnostic biomarker development (experiment 1), and **B.** Validation in experiment 2 with classification thresholds developed from experiment 1. **C.** Venn diagram showing participant overlap in the two experiments (CC: Participants with a history of congenital dense bilateral cataracts and subsequent vision restoration, DC: Participants with a history of bilateral developmental cataracts and subsequent vision restoration).

In experiment 2, used for validation, 15 participants with a history of congenital cataracts took part. One of them was born with incomplete cataracts and therefore likely had some limited pattern vision experience and was therefore excluded. The remaining 14 CC participants were on average 17.07 years old (range = 6 – 39 years). Three of the CC participants in the second experiment were female and 1 was left-handed. The geometric mean visual acuity of the CC participants in the second experiment was 0.229 (decimal, range = 0.051 – 0.7), and their mean age at surgery was 42.14 months (range = 1 month – 17.75 years). The mean time since surgery in the CC group in the second experiment was 13.92 years (range = 3.33 – 37 years). The DC group of this experiment consisted of 16 individuals. Data of one of these participants were left out of the analysis due to a history of seizures and developmental delay. The remaining 15 DC participants had a mean age of 14.47 years (range = 9 – 24 years), and a geometric mean visual acuity of 0.619 (decimal, range = 0.23 – 1.00). This was significantly higher than the visual acuity of the CC group, expressed in LogMAR units (one-tailed *t-*test, *t*(22.278) = 4.693, *p* < .001). The age of the DC participants, however, was not significantly different from the CC participants, two-tailed *t-*test, *t*(16.696) = 0.908, *p* = .377. Four participants were female, and one was left-handed. The mean age at surgery for the DC group was 7.36 years (range = 1.92 – 14.42 years), and the mean time since surgery was 7.52 years (range = 4.58 – 22 years). As in the first experiment, a group of participants matched for age, sex, and handedness to the cataract-reversal participants took part in the study (*N* = 29, mean age = 15.66 years, range = 7 – 38 years, 7 female, 2 left-handed). All of them had normal or corrected-to-normal vision and no history of sensory problems. The second experiment had 3 new CC and 7 new DC individuals who did not partake in the first experiment, that is, 34% new individuals with a history of cataracts (see Fig. 1). Five control participants who took part in experiment 1 took part in experiment 2 as well, and the rest (83%, 24 out of 29) were new.

All CC and DC participants were recruited and tested at the L V Prasad Eye Institute, Hyderabad, India (LVPEI). Control participants were from the local community of Hamburg (see Fig. 1 for participant flow diagram according to the *Standards for Reporting of Diagnostic Accuracy, STARD*). For taking part in the study, adult participants received a small monetary compensation and minors received a present. The study was approved both by the local ethics commission of the faculty of Psychology and Human Movement at the University of Hamburg and the institutional ethics review board of LVPEI, and conformed to the ethical principles outlined in the Declaration of Helsinki (2013).

### 2.2 Procedures

**Fig. 2:**
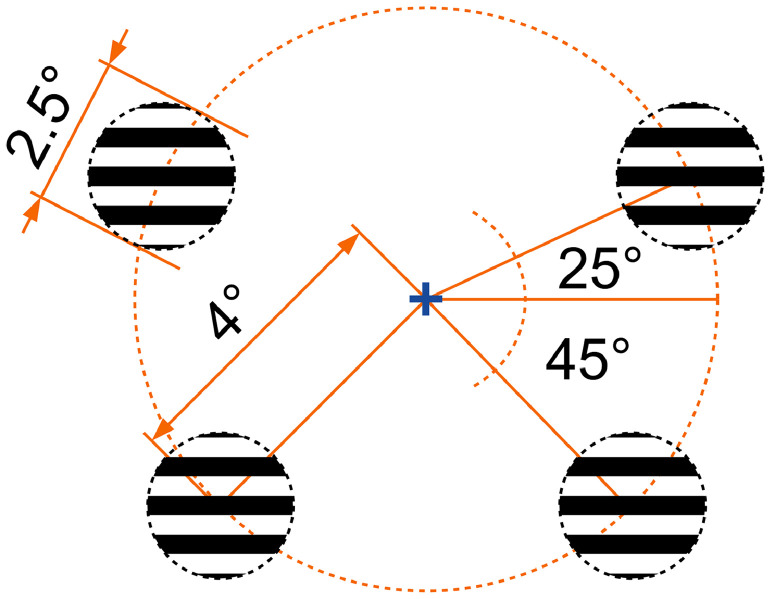
Visual stimulus locations used in the two experiments. The stimuli were presented randomly and one at a time, with rare vertical gratings serving as behavioral targets.

In both experiments, all participants were presented with circular visual stimuli subtending a visual angle of 2.5° presented in one of the four quadrants of the visual field on a gray background as depicted in Fig. 2. The eccentricity of the center of the stimuli was 4° and the stimuli consisted of either horizontally (*P* = .80) or vertically oriented (*P* = .20) monochromatic square wave grating patterns. The spatial frequency of the gratings was 2 cycles/degree, which was within the range of spatial frequencies reported to be affected least in congenital cataract reversal individuals (0.33 – 2 cycles/degree) [28]. The stimuli were presented for a duration of 150 ms with a mean interstimulus interval of 1.85 s (range = 1.5 – 2.2 s). The locations were chosen to optimally stimulate opposing locations in the upper and lower banks of the calcarine sulcus and are widely used in studies investigating retinotopic processing in humans [25]. VERPs elicited by pattern *onset* stimuli as employed in the current study, in contrast to VERPs elicited by pattern *reversal* stimuli, have been reported not to be suppressed by nystagmus [29,30]. This aspect makes them especially suitable for being employed in the CC population, as nystagmus is one of the common sequelae associated with (transient) congenital visual deprivation [4,5].

Participants responded to the infrequently presented vertically oriented patterns using a foot pedal in experiment 1 and with a mouse in experiment 2 (for details, see Supplementary Materials *S2: Behavioral Data Analysis*). Two CC participants in experiment 1, and 2 DC participants and 1 CC participant in experiment 2 were not able to discriminate the grating orientations and observed the stimuli passively. The ability of these participants to accurately perceive the quadrant of stimulus appearance was tested in all cases. Before the commencement of the experiment, participants received a practice block. The stimuli were presented at the L V Prasad Eye Institute on a Dell IN2030 Monitor and at the University of Hamburg on a Samsung P2370 monitor, both of which had a refresh rate of 60 Hz and a nominal luminance value of 250 cd/m^2^. In the first experiment, the participant sat at a distance of 45 cm from the monitor and in the second, at a distance of 65 cm. The angular measurements were, however, exactly the same. For stimulus presentation and EEG triggering we used the PsychoPy framework (version 1.83) [31]. Both experiments comprised additional stimuli not analyzed here. Part of the data from experiment 1 have been published elsewhere [12].

For EEG data acquisition, a custom cap from EASYCAP was used with Ag/AgCl electrodes with a left earlobe reference (EASYCAP GmbH, Herrsching, Germany), and average referenced offline. The recording locations, based on the standard 10/20 system, were the following: FP1, FP2, F7, F3, Fz, F4, F8, FC5, FC1, FCz, FC2, FC6, T7, C3, Cz, C4, T8, TP9, CP5, CP1, CP2, CP6, TP10, P7, P3, Pz, P4, P8, O1, O2, F9, and F10. EEG data were recorded using BrainVision BrainAmp DC/MR Amplifiers (Brain Products GmbH, Gilching, Germany), through an online bandpass filter with cutoff frequencies of 0.016 Hz and 250 Hz, operating at 1 kHz sampling rate. Offline, the data were notch-filtered for electrical line noise artefacts at 50 Hz and its harmonics if sharp peaks were present at the frequency spectrum of the data. Following this, biological artefacts related to blinks, saccades, heartbeats and excessive muscle activity were removed with an independent components analysis using the EEGLAB framework running on MATLAB (EEGLAB version: 11.5.4b, MATLAB version 2012b; MathWorks, Natick, MA) [32]. Before averaging, EEG epochs with blinks or eye movements in the time window of −25 ms to 175 ms with respect to the stimulus onset were marked for rejection to prevent potential VERP reductions caused by eye movements or blinks during stimulus presentation. For this purpose, we analyzed the separated blink and eye movement independent components, if present, using their stereotypical topographies [33,34]. The maximum of five times the standard deviations of the frontopolar electrodes FP1 and FP2 provided the threshold value for detecting blinks, and the maximum of 3 times the standard deviations of the electrodes F9 and F10 were used as the threshold of detecting eye movements. In the separated blink and eye movement independent components data, that is, data containing the major ocular artefacts, signal values exceeding the absolute value of these thresholds were rejected. The data were then epoched from 1000 ms prior to 1000 ms after the onset of visual stimulation after average referencing and low-pass filtering with a cut-off frequency of 40 Hz. To increase power, for EEG trials generated by left visual field stimulation, we swapped the data of each electrode with the data of the electrode in its mirrored location with respect to the nasion-inion axis, leaving the midline electrodes untouched, as was used in already published work [12]. To exclude contamination from motor artefacts from possible responses, we rejected epochs which contained a button response within 500 ms of the stimulus onset for participants who operated the response devices themselves. Finally, visual event-related potentials (VERPs) were calculated by averaging the trials for each time point, yielding two VERPs per participant, one for upper visual field and one for lower visual field stimuli. In experiment 1, the mean number of artifact-free trials for the upper visual field stimulus condition was 128 (*SE* = 5.106), with a minimum value of 40 trials. In experiment 2, the mean number of artifact-free trials from upper visual field stimulation entering the analyses was 81 (*SE* = 4.539), with a minimum of 21 trials.

### 2.3 Biomarker development and statistical analysis

We used data exclusively from the first experiment for the development of the P1 wave based biomarker for congenital visual deprivation. To determine the P1 wave latency, in each single participant and condition (upper and lower visual fields), we first identified the largest P1 wave peak separately at each of the 13 electrodes posterior to the midline (TP9, CP5, CP1, CP2, CP6, TP10, P7, P3, Pz, P4, P8, O1, and O2) in a 100 – 200 ms post-stimulus time window using the *findpeaks* function in MATLAB. The latency for an individual and condition was determined at the electrode at which the highest P1 wave peak was observed. To prevent detecting spurious peaks found by the algorithm, we calculated the mean P1 amplitude in a 50 ms window centered at each individual's latency. Five participants, all of them CC, displayed a negative value for the average P1 amplitude for both upper and lower visual field stimulation in this window – a numerical absence of the P1 wave – and were therefore excluded from latency calculations. Thereafter, for each of the three groups (CC/DC/Control), we separately calculated the average P1 wave latency for the upper and lower visual field stimulus conditions. The average latency of the P1 wave over all the three groups and both stimulus locations was 145 ms (range: 139 – 155 ms). In the typically sighted controls, we ran a two-tailed cluster-based permutation test using *FieldTrip* software over the 13 electrodes posterior to the midline (where the P1 wave is most discernible) and a latency range of 50 ms centered at this P1 peak latency to test whether visual field location (upper vs. lower) had an effect on the P1 amplitude [35]. The cluster-based permutation test revealed that P1 amplitudes elicited by upper visual field stimuli were significantly larger than for lower visual field stimulations (10,000 permutations, *p* < .001). We therefore used VERPs to upper visual field stimuli for the development of the biomarkers.

**Fig. 3:**
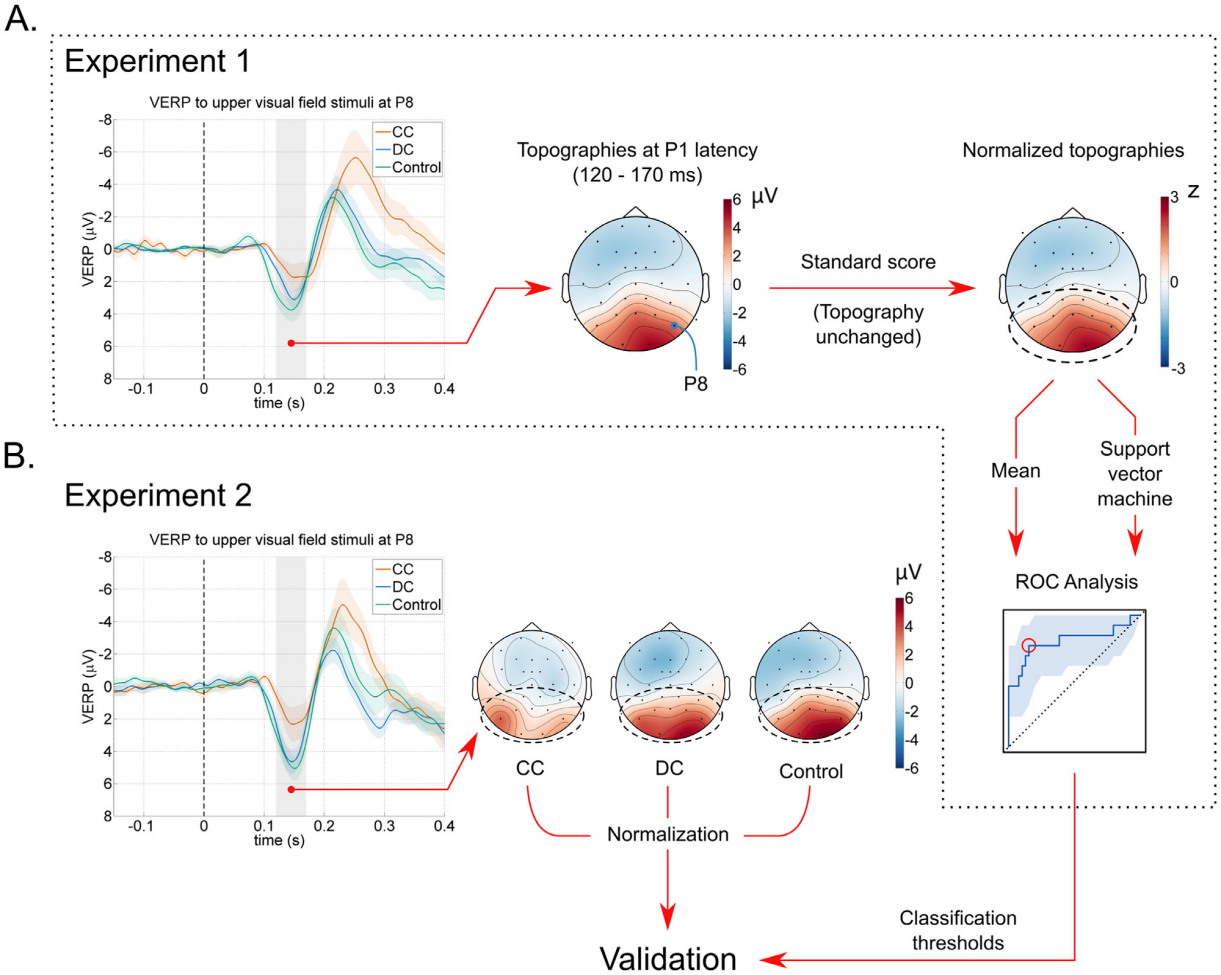
Development and validation of an electrophysiological biomarker for classifying sight recovered bilateral congenital vs. bilateral developmental cataract individuals. **A.** Visual event-related potentials (VERPs) to upper visual field stimuli from experiment 1 were used for classifier development. The VERPs at electrode P8 is depicted for all three groups. Shaded colored areas show the standard error of the mean. We first extracted the mean P1 amplitude over a 50 ms time range around the average P1 latency (note that electrodes below the nasion-inion “great circle” are depicted outside the head circumference). Thereafter, we normalized the P1 amplitude over the whole electrode montage and calculated the mean value over the posterior electrodes as a biomarker (Mean posterior P1, MPP1). We created an additional biomarker by employing a linear support vector machine to find the optimum linear coefficients for classifying CC individuals (SVMP1). Receiver operating characteristics analysis was used to find optimum threshold values, maximizing the *Youden's J* parameter. **B.** Validation of the classification algorithm. VERPs to upper visual field stimuli were obtained from a separate experiment using a different viewing distance. The biomarkers MPP1 and SVMP1 for congenital cataract history were obtained for each individual and classification performance was measured using the thresholds obtained from the first experiment.

As a first step for developing a P1-based biomarker, for each individual participant we extracted the mean of the VERP amplitude in the range of 120 – 170 ms, that is, in a 50 ms period centered at the mean P1 peak. VERP amplitudes in children are considerably higher than in adults, but the topography of ERP waves have been reported to be remarkably stable across age groups from the 4th year of life [36]. To remove age as a confounding factor, we converted the mean P1 amplitude to standard scores calculated across all electrodes, a topography-preserving procedure that normalizes amplitude [37]. Thereafter we developed two biomarkers from the standardized P1 wave topography. The first, the mean posterior P1 biomarker (MPP1), was obtained by simply taking the mean of the normalized P1 wave amplitude over the 13 posterior electrodes (TP9, CP5, CP1, CP2, CP6, TP10, P7, P3, Pz, P4, P8, O1, O2; see Fig. 3). To verify the reduction of P1 amplitudes in the CC group, we submitted the MPP1 values to an ordinary least squares linear model with MPP1 values as the dependent variable and *Group* (CC/DC/Control) as the categorical predictor in the R programming environment, version 3.6.1 [38]. For this analysis, the control group was set as the reference group.

**Fig. 4:**
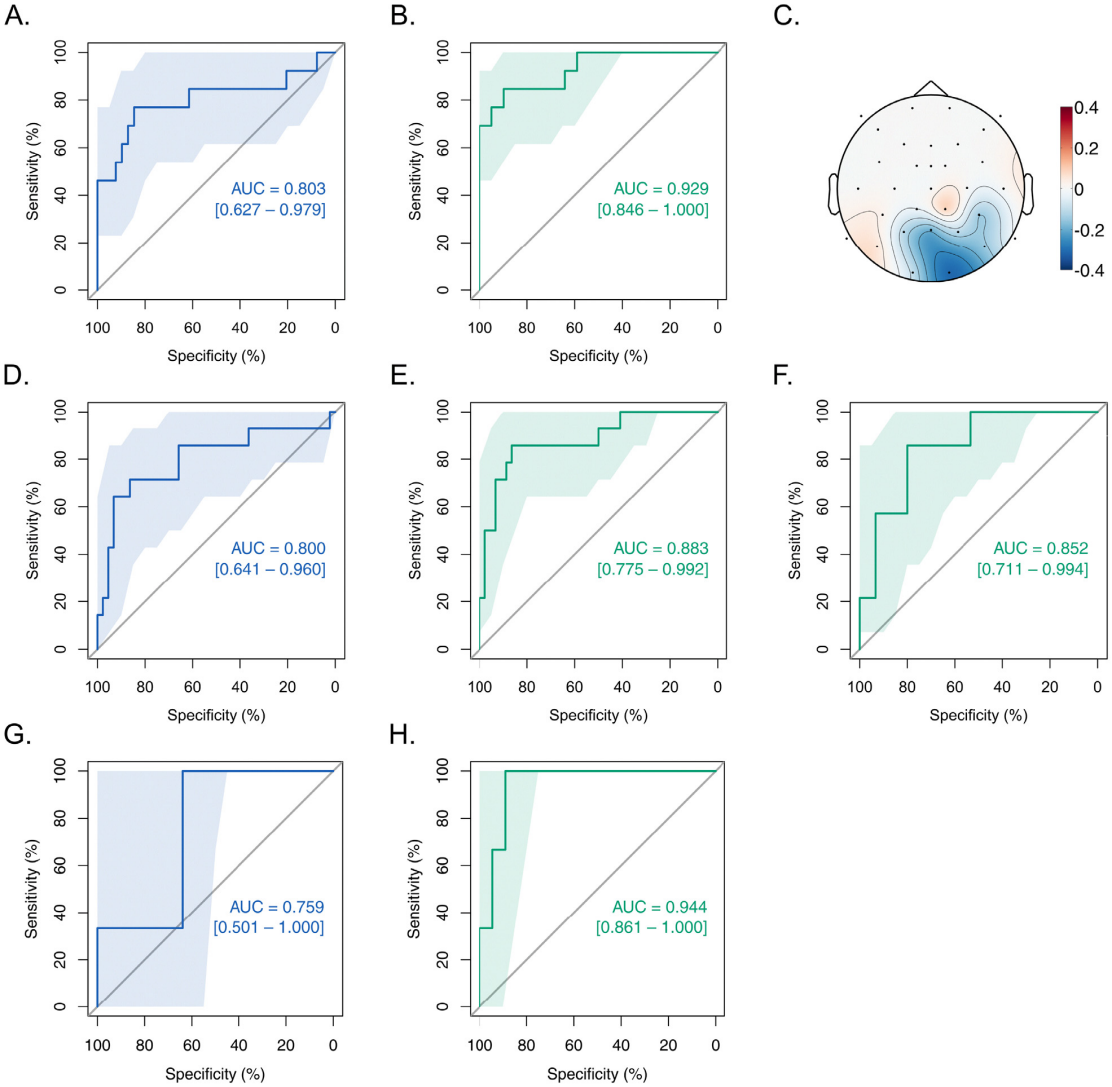
Development and validation of the P1-based biomarkers for classifying congenital cataract individuals. **A.** Receiver operating characteristic (ROC) curve from the first experiment involving all three groups classifying CC individuals using the MPP1 marker. **B.** ROC curve from experiment 1 for the SVMP1 marker. **C.** Optimal linear weights for the posterior electrodes for SVM-based classification, plotted as a topography (unitless). **D.** and **E.** ROC curves for MPP1 and SVMP1 based classification for experiment 2. **F.** ROC curve for the SVMP1 validation performance in experiment 2 with weights developed from a group of only CC and DC individuals in experiment 1, applied for the combined group of only CC and DC individuals in experiment 2. **G.** and **H.** ROC curves for experiment 2 as in panels D. and E., but with only the new CC and DC participants (*N*_CC_ = 3, *N*_DC_ = 10, *N*_Control_ = 29). Each panel displays the areas under the ROC curves (AUCs) and their 95% confidence intervals (CIs). CI bands for sensitivities are depicted as shaded areas (all sensitivity CIs obtained with stratified bootstraps, *n* = 2000). All *p*s < .05 for the ROCs. For statistical details see Table 1.

The development procedure for the MPP1 biomarker was equivalent to weighting each of the posterior electrodes by a factor of $\frac{1}{13}$ and performing a sum. To explore whether other linear weighting strategies might lead to a classification improvement, we additionally submitted the normalized P1 wave amplitude values from the posterior electrodes of all participants from the three groups (CC/DC/Control) to a linear support vector machine (SVM) returning the optimum weight for each electrode for classifying CC individuals (see Fig. 4, panel C) [39,40]. The optimum value for the *cost* hyperparameter of the linear SVM-based biomarker was derived through grid search using the *e1071* library, version 1.7–2 [41], in the R programming environment, version 3.6.1 [38]. This classifier derived from a linear support vector machine was termed SVMP1. Both the MPP1 and the SVMP1 biomarkers were submitted to receiver operating characteristics (ROC) analyses for determining the optimum cut-off point for the classification, maximizing the *Youden's J* parameter which indicates the vertical distance from the diagonal chance performance line (see Fig. 4) [42]. Stratified bootstrap tests (*n* = 2000) were used to compare the area under the ROC curves (AUCs) of the biomarkers. Statistical analyses were performed with the *pROC* package, version 1.15.3 [43], as well as the core packages in the R programming environment, version 3.6.1 [38]. Confidence intervals for the AUCs were calculated employing the DeLong's method [44].

For validation, we exclusively used the VERPs from the second experiment. For each participant taking part in the second experiment, we calculated the MPP1 and SVMP1 classification scores as outlined above. For defining the SVMP1 classifier, the weights derived from experiment 1 were used. Classification performance was assessed by utilizing the thresholds obtained from the first experiment to classify the MPP1 and SVMP1 values of the second experiment. We additionally developed and validated the classification in a combined group of only CC and DC individuals excluding the typically sighted controls, since the most conservative and crucial scenario involves classification of CC from DC individuals.

To examine potential effects of visual recovery period as well as age at surgery on the classification accuracy, we ran four logistic regression models, two for each experiment, with classification success from the MPP1 or the SVMP1 biomarker as the dependent variable, and time since surgery (in months, normalized) and age at surgery (in months, normalized) as the independent variables. Without scaling (or more appropriately, centering) the independent variables, the intercept from the logistic regression model would provide the log likelihood of accurate responses when the independent variables, mean age at surgery (months) and mean time since surgery (months), were both zero, which cannot be directly interpreted. After scaling the independent variables, the intercept in this model provided the classification accuracy at the mean age of surgery and the mean duration of visual recovery, and the regression coefficients represented an estimate of the departure from this level of accuracy as a function of age at surgery or time since surgery (in months).

The article adheres to the STROBE guidelines [45].

## 3 Role of the funding sources

The funding sources did not play any role in the study design, data collection, data analysis, interpretation of the data, writing of the article or in the decision to submit the article for publication except by providing financial support to fund the scientists and research. The corresponding author had full access to the collected data and had final responsibility for the decision to submit for publication, with the consent of all co-authors.

## 4 Results

In the first experiment, used for developing the electrophysiological biomarkers, employing an ordinary least squares linear model we found that normalized posterior P1 amplitude (MPP1) values were significantly different across groups, *F*(2, 49) = 14.591, *p* < .001, $R_{\text{adj}}^{2}$ = .348. While the DC group was not significantly different from the control participants (*β*_DC_ = −0.137, 95% CI = [−0.430, 0.156], *SE*_DC_ = 0.146, *p* = .351), the CC group exhibited a very large [46], statistically significant reduction of MPP1 values, *β*_CC_ = −0.777, standardized *β* = −1.462, 95% CI = [−1.070, −0.484], *SE*_CC_ = 0.146, *p* < .001. The very large reduction of the normalized posterior P1 amplitudes in the CC group was replicated in experiment 2, *F*(2, 55) = 12.479, *p* < .001, $R_{\text{adj}}^{2}$ = .287, *β*_CC_ = −0.654, standardized *β* = −1.354, 95% CI = [−0.919, −0.388], *SE*_CC_ = 0.133, *p* < .001. The DC group was once more indistinguishable from the control participants, *β*_DC_ = −0.112, 95% CI = [−0.372, 0.148], *SE*_DC_ = 0.130, *p* = .391.

The ROC analysis involving all three groups indicated that both MPP1 and SVMP1 classifiers exhibited excellent performance for identifying congenital pattern vision deprivation (areas under the ROC curve, AUCs > 0.8, see Fig. 4 and Table 1: Development) [47]. The SVMP1 biomarker showed a higher performance than the MPP1 biomarker (stratified bootstrap test, *D* = −2.330, *n* = 2000, *p* = .020, see also Table 1: Development). This was not unexpected, since the SVM method utilized a large number of variables (13 electrodes) to classify the CC participants. The optimal weights for the linear SVM-based classification are depicted as a topography in Fig. 4, panel C. The optimum threshold maximizing the *Youden's J* parameter for the MPP1 classifier was 0.408 (z-score) [42], and for the SVMP1 classifier the optimum threshold was −0.830 (linear weighted z-score).

**Table 1:** Classifier performance. For the mean posterior P1 (MPP1) and the linear support vector machine based P1 (SVMP1) biomarkers, key classification parameters for the development and validation are shown: Area under the ROC curve (AUC), sensitivity, specificity, and likelihood ratios.

**Development (Experiment 1)**					
	AUC(95%CI)	Sensitivity	Specificity	Positive Likelihood Ratio	Negative Likelihood Ratio
MPP1	0.803 (0.627 – 0.979)	0.769	0.846	4.99	0.27
SVMP1	0.929 (0.846 – 1.000)	0.846	0.897	8.21	0.17
**Validation (Experiment 2. Sensitivity and specificity obtained using the classification threshold values generated from Experiment 1)**					
	AUC(95%CI)	Sensitivity	Specificity	Positive Likelihood Ratio	Negative Likelihood Ratio
MPP1	0.800 (0.641 – 0.960)	0.643	0.909	7.07	0.39
SVMP1	0.883 (0.775 – 0.992)	0.786	0.864	5.78	0.25
					
**Validation with only CC and DC individuals in Experiment 2. Sensitivity and specificity obtained using thresholds from Experiment 1 with only CC and DC individuals**					
	AUC(95%CI)	Sensitivity	Specificity	Positive Likelihood Ratio	Negative Likelihood Ratio
MPP1	0.757 (0.568 – 0.946)	0.643	0.800	3.22	0.45
SVMP1	0.852 (0.711 – 0.994)	0.857	0.800	4.29	0.18

A validation of the ROC analyses with data from experiment 2 of all three groups (CC/DC/Control) using classification thresholds obtained from experiment 1 indicated a reliable classification performance for both the MPP1 and the SVMP1 classifiers (AUCs > 0.8, see Table 1: Validation). Importantly, both classifiers exhibited excellent specificity (90.9% for the MPP1 and 86.4% for the SVMP1 biomarker). The SVMP1 biomarker exhibited a better classification performance than the MPP1 biomarker, indicated by a significantly higher AUC of its ROC curve (stratified bootstrap test, *D* = −1.989, *n* = 2000, *p* = .047). Moreover, keeping only the sight recovery participants who took part in experiment 2 but not in experiment 1 (*N*_CC_ = 3, *N*_DC_ = 10, *N*_Control_ = 29) impressively demonstrated the reliability of both biomarkers (see Fig. 4, panels G and H). Compared to MPP1 biomarker, AUC = 0.759, 95% CI = [0.501 – 1.00], the SVMP1 biomarker performed better, AUC = 0.944, 95% CI = [0.861 – 1.00], stratified bootstrap test with 2000 replicates, *D* = −1.980, *p* = .048. Using the threshold value from experiment 1, in this conservative test all of the 3 new CC participants in experiment 2 were detected by the SVMP1 biomarker with a sensitivity of 100%, while retaining a high specificity of 88.89%.

We additionally tested and verified the performance of the MPP1 and the SVMP1 in a group comprising only the CC and the DC individuals. The validation tests in experiment 2 indicated a higher performance of the SVMP1 classification method (*N*_CC_ = 14, *N*_DC_ = 15, sensitivity = 85.7%, specificity = 80.0%) compared to the MPP1 (sensitivity = 64.3%, specificity = 80.0%), but statistically only a trend for a higher AUC was observed (stratified bootstrap test, *D* = −1.874, *n* = 2000, *p* = .061; see Table 1: Validation with only CC and DC individuals). At an individual level, a sensitivity of 85.7% (=12/14) signifies that only 2 of the 14 CC participants were misclassified. Likewise, a specificity of 80.0% (=12/15) means that only 3 of the 15 DC participants were misclassified by the SVMP1 classifier. The accuracy achieved was 82.8%.

The logistic regression models ascertained that classification accuracy depended neither on the age at surgery nor on the period of visual recovery: A statistically significant intercept term in both experiments suggested that the classification accuracies for the mean age at surgery and mean visual recovery period were significantly above chance level (Exp. 1: *β*_MPP1_ = 1.367, *SE* = 0.535, *p* = .011; *β*_SVMP1_ = 1.604, *SE* = 0.575, *p* = .005, Exp. 2: *β*_MPP1_ = 1.108, SE = 0.472, *p* = .019; *β*_SVMP1_ = 1.160, *SE* = 0.440, *p* = .008). The independent variables, normalized age at surgery and normalized time since surgery, on the other hand, did not have significant coefficients for any of the biomarkers in any of the two experiments (Exp. 1: *p*s > .160, Exp. 2: *p*s > .158).

Finally, we observed excellent to almost perfect inter-test classification agreement for the participants who took part in both experiments (Cohen's *κ =* 0.829, see Supplementary Materials *S4. P1-Based Biomarkers Exhibit High Test-Retest Reliability*) [48,49].

## 5 Discussion

In two separate experiments involving visual event-related potentials (VERPs) elicited by simple, high contrast visual grating stimuli presented in different quadrants of the visual field, we aimed to develop and validate an electrophysiological biomarker for the classification of sight-restored patients who had suffered a transient phase of bilateral cataracts, either congenital or developmental. Based on previous reports of a reduced P1 amplitude in the VERPs of sight-restored congenital but not developmental cataract patients [9,12,24], we tested whether the P1 amplitude as a marker of extrastriate cortical processing is capable of classifying vision-restored congenital cataract (CC) patients and distinguishing them from vision-restored developmental cataract (DC) patients [25]. Two classification scores were derived from the P1: (1) The mean P1 amplitude over posterior electrodes after normalizing the scalp topography (MPP1), and (2) a weighted sum of the normalized posterior P1 amplitudes derived from a linear support vector machine (SVMP1). We made use of the detailed information listed in the medical records of the participating cataract patients (see *Method: Participants*) for labeling their etiologies. Importantly, these criteria did not involve EEG-based parameters. The classification results strongly suggest that the P1 amplitude is a robust electrophysiological biomarker for classifying congenital cataract-reversal individuals, even after extensive recovery periods since cataract removal surgery (at least one year and up to 37 years in the present study sample, see also *Methods: Participants).*

Extensive functional reorganization of the extrastriate visual cortex has been reported in vision-deprived non-human primates tested after a short period (1 – 3 weeks) of visual recovery [26]. The present results suggest that congenital blindness in humans leads to irreversible changes in the neural systems beyond the primary visual cortex, which are discernible at an individual level using noninvasive and readily accessible electrophysiological methods after sight restoration and a substantial period of vision recovery. By contrast, using the first cortical VERP (C1 wave) we have recently demonstrated evidence for a retinotopic activation of early visual cortex in sight-restored CC individuals. The C1 onset latencies were indistinguishable between CC patients and their matched controls, as in the DC group [12]. A by and large preserved if at all enhanced C1 (difference) wave but markedly reduced P1 wave excludes trivial accounts for the high performance of P1-based biomarkers, such as poor fixation due to inadequate attention or nystagmus. In fact, a classification of CC individuals based on the normalized C1 wave amplitudes or the C1 difference waves was not possible (See section S3, *Supplementary Materials*). The failure to classify a history of congenital pattern vision deprivation based on the first visual cortical (difference) potential, in contrast to the success of the P1-based biomarkers, additionally indicates that the P1-based biomarkers were likely not driven by a general reduction of the VERP in the CC group e.g. caused by nystagmus or lack of fixation. To the best of our knowledge, the present study is the first one reporting an electrophysiological classification process for CC vs. DC individuals, or more generally, for sight recovery individuals with congenital vs. late onsets of visual impairments.

The persistent P1 wave reduction in sight recovery individuals, observed following transient congenital pattern vision deprivation, might have been caused by either a generally attenuated response from extrastriate visual cortical circuits and/or by a lack of synchrony of the elicited P1 wave across trials, leading to a reduced mean P1 amplitude. Recent research on strabismic as well as anisometropic amblyopia has argued that a trial-to-trial latency difference of P1 activity might underlie a similar reduction of P1 wave amplitude observed in the amblyopic eye compared to the fellow eye [50]. Visual experience seems to be necessary for the temporal tuning of extrastriate cortex activity. Thus, the P1 reduction in CC individuals in the present study might be driven by a similar mechanism. Moreover, the P1 wave has been associated with alpha-wave related inhibitory activity in the visual cortex [51]. According to the *P1 inhibition timing* hypothesis, the alpha wave phase at the presentation of a stimulus influences the P1 wave, reflecting inhibition-related activity involved in suppressing task-irrelevant neural circuits [52]. In fact, the P1 wave observed in the present study was larger over task-irrelevant ipsilateral scalp sites (see Fig. 3), in accord with this hypothesis. Additional evidence for compromised alpha oscillatory activity in CC individuals suggests that an altered excitatory-inhibitory balance might be contributing to the P1-wave related biomarkers [13,53], in line with non-human animal research indicating persistent changes in inhibitory networks in response to binocular visual deprivation [54].

The electrophysiological diagnostic approach developed in the present study could augment existing methods for classifying congenital from developmental cataract-reversal individuals, and possibly more generally, transient congenital from transient developmental visual deprivation. This could be of considerable value for establishing an etiology in the absence of reliable information from other sources, or in the presence of symptom overlap at late presentation. Childhood cataract has been reported to cause about 30% of all avoidable childhood blindness cases in low-income countries, where many of the late-treated individuals reside [1,14]. The procedure developed in the present study is highly specific and thus will aid prognostic estimates in patients consulting and rehabilitation effort planning. For example, a strong reduction of the P1 wave in a post-operative individual might indicate the need for more extensive rehabilitation strategies for CC than for DC individuals after sight restoration. The issues associated with rehabilitation of sight restored CC individuals go beyond the treatment of visual acuity problems. For example, patients with a history of bilateral congenital cataracts are more likely to suffer from face and motion processing related deficits compared to individuals who had suffered from a period of transient unilateral congenital visual deprivation, or bilateral visual deprivation later in childhood [9,11,13,17]. Moreover, multisensory integration is partially compromised in sight restored CC individuals, and there is evidence for an interference of coexisting auditory input on visual processing [55], [56], [57]. Thus, proposed multisensory rehabilitation approaches might well work for sight restored DC patients but visual rehabilitation strategies addressing different levels of visual processing might work best for sight restored CC patients [58].

Furthermore, the P1-based biomarker could aid clinical as well as basic research. Outcomes of childhood cataract surgery, postsurgical treatment, and rehabilitation planning are areas of active clinical research [19,20], and these outcomes critically depend on cataract etiology, interacting with the duration of transient blindness [4]. An otherwise highly effective treatment (e.g. surgical procedure) might show diverging outcomes in CC and DC patients. Thus, the treatment would be evaluated differently depending on the proportion of CC and DC patients in the sample. In fact, many investigations on childhood cataract to date have not distinguished between these two distinct patient groups [14], thus increasing the probability of confounding outcomes and invalid conclusions, in particular severely affecting studies spanning multiple centers in different countries where congenital and developmental cataracts have different prevalences. A P1-based electrophysiological marker would contribute to better stratify distinct patient groups and would therefore help reducing error variance emerging from averaging across qualitatively distinct clinical groups.

For basic research aiming at identifying the genetic and experience dependence of functional and structural brain development, a reliable distinction between CC and DC patients is essential [59]. Differences in neural and associated behavioral measures between patients with a congenital vs. a late onset of transient blindness are interpreted as evidence for sensitive periods in brain development, that is, the crucial role of early experience for the typical development of neural circuits. Neuroscience research in cataract-reversed patients is one of the rare opportunities to investigate the neural mechanisms associated with sensitive periods in humans [60], [61], [62]. The results obtained from these populations allow, on the one hand, understanding the role of visual experience in driving typical development. On the other hand, these findings are not only important for improving treatment of patients with sensory deficits, but additionally for any type of program aiming at remediating aberrant early sensory experience later in life. The present study in CC vs. DC individuals was possible due to access to detailed medical records with pre- and post-surgery assessments of the eyes, family reports and family history. However, in many if not most settings, especially in developing countries, such detailed patient records are most of the time unavailable. Therefore, a standardized electrophysiological classification method as suggested in the present study would greatly aid patient group assignment across different centers and labs. How early the P1 classification procedure is feasible after surgery, however, still needs to be established. All sight recovery individuals in the present study were tested after at least one year following surgical intervention, likely providing considerable time for visual recovery. The time course of P1 wave development in DC individuals after surgery is crucial for the assessment of the biomarkers in a longitudinal setting. If a typical P1 wave is observed in DC individuals immediately after surgery, as the reduced P1 amplitude in CC individuals likely indicates impaired extrastriate cortical development, we predict that P1 based classification will work as soon as the patient can be tested post-surgery. If the P1 wave conversely develops over a longer post-surgical time course, systematic associations between the P1-based biomarkers and behavioral measures will possibly offer informative electrophysiological correlates of visual system recovery. In addition, developmental cataract reversal individuals with a history of early-onset, dense bilateral cataracts could eventually help pinpointing the closure of the sensitive period for a typical P1 wave development. In the present study, the accuracy of the biomarkers did not depend on the age at surgery, or time since surgery, suggesting a high stability of the P1 impairment in CC individuals. Moreover, the DC groups in both experiments exhibited normalized posterior P1 amplitudes which were indistinguishable from the typically sighted controls, suggesting the existence of a sensitive period after which pattern vision deprivation does not reduce the P1 amplitude. While we predominantly tested late treated congenital cataract patients (> 5 months of binocular deprivation) except in 4 cases in experiment 1 and 5 cases in experiment 2, it remains an open issue how many weeks of binocular deprivation must persist to cause reliable P1 amplitude reductions [10].

Finally, the robust performance of the biomarkers when tested in a group of only CC and DC individuals excluding the control participants, who were tested in Germany, excludes factors related to ethnicity or culture driving the high classification performance.

In conclusion, the present study obtained and validated a reliable and specific electrophysiological biomarker for classifying sight recovery patients with a history of bilateral congenital vs. developmental cataracts, which on the one hand will allow for tailored treatment and rehabilitation efforts, and on the other hand will aid clinical and basic research by reducing error variance due to averaging across data from patients with heterogeneous etiologies.

## 6 Data statement

The period of recruitment, data collection, and data analysis was 2015 – 2020. Anonymized data and materials will be made available to external investigators upon reasonable request to the corresponding author through data transfer agreements approved by the stakeholders, under stipulations of applicable law including but not limited to the General Data Protection Regulation (EU 2016/679).

## Declaration of Competing Interests

All authors report grants from the German Research Foundation (DFG) and the European Research Council (ERC) during the conduct of the study.

## Appendix Supplementary materials

Image, application 1

Image, application 2

Image, application 3
