# Supplementary material for: An electrophysiological biomarker for the classification of cataract-reversal patients: A case-control study
Source: eClinicalMedicine. 2020 Oct 6;27:100559. doi: 10.1016/j.eclinm.2020.100559 (PMC7548424; doi:10.1016/j.eclinm.2020.100559)
Supplement: Supplementary file 1 [file mmc1.pdf]

Supplementary Materials

An Electrophysiological Biomarker for the Classification of Cataract-Reversal Patients: A Case-Control Study

Suddha Sourav, Davide Bottari, Idris Shareef, Ramesh Kekunnaya, and Brigitte Röder

S1. Participant Characteristics

Sight recovery individuals with a history of congenital bilateral dense cataracts and subsequent surgery (CC) are denoted as CC – XXX. Sight recovery individuals with a history of developmental bilateral cataracts and subsequent surgery (DC) are denoted as DC – XXX.

Table S1: Participant characteristics for the sight recovery individuals with a history of congenital bilateral dense cataracts and subsequent surgery (CC) in experiment 1

Participant ID	Group	Age (yrs.)	Duration of blindness (mo.)	Time since surgery (mo.)	Sex	Handedness	Visual Acuity (Decimal)	Visual Acuity Prior to Surgery (Better Eye)	Nystagmus	Strabismus	Family History
CC – 001	CC	26	5	308	M	Right	0.180	NA	Yes	Esotropia	No
CC – 002	CC	37	24	428	M	Right	0.400	NA	Yes	Esotropia	Yes
CC – 003	CC	11	7	124	M	Right	0.430	Fixates and follows light	Yes	Exotropia	No
CC – 004	CC	13	4	158	M	Right	0.130	Fixates and follows light	Yes	Exotropia	No
CC – 005	CC	33	72	330	M	Right	0.051	NA	Yes	No	Yes
CC – 006	CC	23	4	279	M	Left	0.130	NA	Yes	Esotropia	Yes
CC – 007	CC	13	15	150	M	Right	0.500	Fixates and follows light	Yes	Exotropia	Yes
CC – 008	CC	8	1	100	M	Right	0.700	Fixates and follows light	No	No	Yes
CC – 009	CC	10	11	116	M	Right	0.240	Fixates and follows light	Yes	Esotropia	No
CC – 010	CC	18	25	198	F	Right	0.310	Fixates and follows light	Yes	Esotropia	Yes
CC – 011	CC	19	213	25	M	Right	0.200	Counting fingers at 0.5 m	Yes	Esotropia	Yes
CC – 012	CC	11	42	89	F	Right	0.450	Fixates and follows light	Yes	Exotropia	Yes
CC – 013	CC	10	74	48	M	Right	0.250	0.017	Yes	No	Yes

Table S2: Participant characteristics for the sight recovery individuals with a history of developmental bilateral cataracts and subsequent surgery (DC) in experiment 1

Participant ID	Group	Age (yrs.)	Age at Surgery (yrs.)	Time Since Surgery (mo.)	Sex	Handedness	Visual Acuity (Decimal)	Visual Acuity Prior to Surgery (Better Eye)	Nystagmus	Strabismus	Family History
DC – 001	DC	16	12	53	M	Right	1.000	Counting fingers at 0.5 m	No	No	No
DC – 002	DC	19	14	55	M	Right	1.000	0.200	No	No	No
DC – 003	DC	12	6	63	F	Right	1.000	0.500	No	No	No
DC – 004	DC	11	8	39	F	Right	0.530	0.159	No	No	No
DC – 005	DC	13	8	68	M	Right	0.710	0.100	No	Exotropia	No
DC – 006	DC	12	8	51	M	Right	0.600	0.400	No	No	No
DC – 007	DC	12	6	77	M	Right	1.000	0.250	No	No	Yes
DC – 008	DC	18	12	74	F	Right	0.790	Sees hand movement	No	No	No
DC – 009	DC	16	10	67	M	Right	0.740	0.400	No	No	No
DC – 010	DC	24	2	264	F	Right	0.440	Fixates and follows light	No	Exotropia	No
DC – 011	DC	16	7	110	F	Right	0.530	Counting fingers @ 1 m	No	Exotropia	Yes
DC – 012	DC	20	12	97	M	Right	0.880	0.400	No	No	No
DC – 013	DC	19	17	17	M	Right	0.730	0.200	No	No	No

Table S3: Participant characteristics for the sight recovery individuals with a history of congenital bilateral dense cataracts and subsequent surgery (CC) in experiment 2 (Please note that age of the same participant might vary from Table S1 due to the experiments being run at different time points).

Participant ID	Group	Age (yrs.)	Duration of blindness (mo.)	Time Since Surgery (mo.)	Sex	Handedness	Visual Acuity (Decimal)	Visual Acuity Prior to Surgery (Better Eye)	Nystagmus	Strabismus	Family History
CC – 001	CC	26	5	308	M	Right	0.180	NA	Yes	Esotropia	No
CC – 005	CC	33	72	330	M	Right	0.051	NA	Yes	No	Yes
CC – 014	CC	6	5	70	M	Right	0.240	Not fixating/following light	Yes	No	No
CC – 002	CC	39	24	444	M	Right	0.400	NA	Yes	Esotropia	Yes
CC – 004	CC	14	4	175	M	Right	0.130	Fixates and follows light	Yes	Exotropia	No
CC – 006	CC	24	4	294	M	Left	0.130	NA	Yes	Esotropia	Yes
CC – 007	CC	15	15	166	M	Right	0.500	Fixates and follows light	Yes	Exotropia	Yes
CC – 008	CC	9	1	116	M	Right	0.700	Fixates and follows light	No	No	Yes
CC – 009	CC	11	11	129	M	Right	0.240	Fixates and follows light	Yes	Esotropia	No
CC – 015	CC	8	48	48	F	Right	0.150	Perception of light	Yes	No	Yes
CC – 013	CC	11	74	61	M	Right	0.250	0.017	Yes	No	Yes
CC – 012	CC	11	42	90	F	Right	0.450	Fixates and follows light	Yes	Exotropia	Yes
CC – 011	CC	21	213	40	M	Right	0.200	Counting fingers at 0.5 m	Yes	Esotropia	Yes
CC – 016	CC	11	72	68	F	Right	0.260	Fixates and follows light	Yes	No	No

Table S4: Participant characteristics for the sight recovery individuals with a history of developmental bilateral cataracts and subsequent surgery (DC) in experiment 2 (Please note that age of the same participant might vary from Table S2 due to the experiments being run at different time points).

Participant ID	Group	Age	Age at Surgery (yrs.)	Time Since Surgery (mo.)	Sex	Handedness	Visual Acuity (Decimal)	Visual Acuity Prior to Surgery (Better Eye)	Nystagmus	Strabismus	Family History
DC – 002	DC	19	14	55	M	Right	1.000	0.200	No	No	No
DC – 011	DC	14	7	80	F	Right	0.530	Finger counting at 1 m	No	Exotropia	Yes
DC – 003	DC	13	6	74	F	Right	1.000	0.500	No	No	No
DC – 005	DC	14	8	80	M	Right	0.710	0.100	No	Exotropia	No
DC – 007	DC	12	6	77	M	Right	1.000	0.250	No	No	Yes
DC – 014	DC	12	7	66	M	Right	0.230	0.125	No	No	No
DC – 015	DC	12	7	67	M	Right	0.460	0.100	No	No	No
DC – 009	DC	16	10	67	M	Right	0.740	0.400	No	No	No
DC – 010	DC	24	2	264	F	Right	0.440	Fixates and follows light	No	Exotropia	No
DC – 008	DC	18	12	74	F	Right	0.790	Perceives hand movement	No	No	No
DC – 016	DC	17	12	60	M	Right	0.310	0.160	No	No	Yes
DC – 017	DC	9	2	86	M	Right	0.930	0.154	No	No	No
DC – 018	DC	16	6	113	M	Right	0.520	Finger counting at 1 m	No	No	No
DC – 019	DC	10	3	76	M	Left	0.730	0.065	No	No	Yes
DC – 020	DC	11	1	115	M	Right	0.667	0.154	No	No	No

S2. Behavioral Data Analysis

S2.1. Statistical Analysis of Behavioral Data

In experiment 1, 2 CC participants were not able to discriminate the horizontal vs. vertical grating orientations (i.e. standards from target visual stimuli), although they could fixate and perceive the visual stimulus onsets in different quadrants. Data of these two CC participants and their matched controls were excluded from behavioral analyses. Seven additional CC participants and 4 DC participants performed the task verbally while the experimenter operated the foot pedal for them. Due to a low sample size we did not compare reaction times (RTs) in this experiment but analyzed the hit rates and false positive rates ($N_{CC} = 11$, $N_{DC} = 13$, see Figures S1 and S2). To this end, the hit rates and false positive rates of the CC and the DC groups were compared with those of their matched control groups. We denote the matched control group for the CC group as MCC, and the matched control group for the DC group as MDC. The hit rates and false positive rates were compared using separate one-tailed Welch's *t*-tests based on the reasoning that due to their persistent visual impairments, lower performance (i.e. higher false positive rates and lower hit rates) in the sight recovery groups (CC/DC) could be expected compared to their matched control groups (MCC/MDC). We defined a *hit* as a response following a target stimulus between 155 – 3000 ms after its onset, with no other target stimulus between the onset of the target and the response. A false positive response was defined as a response following a standard visual stimulus between 155 – 3000 ms after its onset, with no other stimulus between the visual stimulus and the response.

In experiment 2, participants responded with a computer mouse. Data of 1 CC participant and 2 DC participants who could not reliably discriminate the grating orientations and observed the stimuli passively were excluded from behavioral analyses along with their matched control participants. As in experiment 1, we ensured that these participants could fixate and perceive the stimulus appearances in different quadrants. Experimenter response coding was used for one further CC participant; we excluded this participant and their matched control from the RT analysis but kept them for the analysis of hit rates and false positive rates ($N_{CC} = 12$ for RT analysis, 13 for hit rate and false positive rate analyses; $N_{DC} = 13$). As in experiment 1, we used one-tailed Welch's *t*-tests, testing for the presence of a slower RT, higher false positive rates, and lower hit rates in the sight recovery groups compared to their control groups matched for age, sex, and handedness (see Figures S3 – S5).

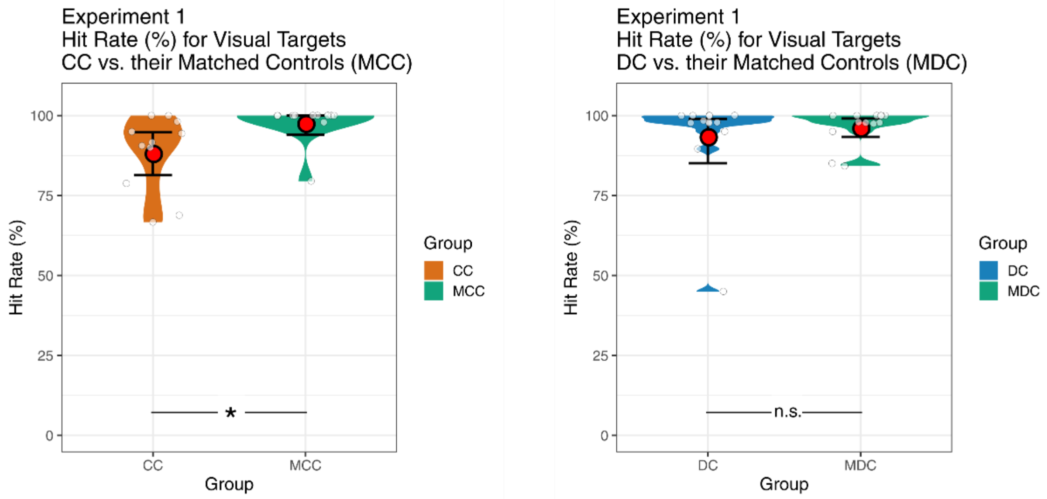

Figure S1. Left: Hit rate (%) for the CC and the MCC group in experiment 1, *N* = 11; Right: Hit rate (%) for the DC and the MDC group in experiment 1, *N* = 13 (∗ : *p* < .05, one-sided Welch's *t*-test). The red circles indicate group means, with the error bars denoting 95% confidence interval obtained using regular bootstrap with 10,000 replicates [1]. White dots are individual data points jittered for readability.

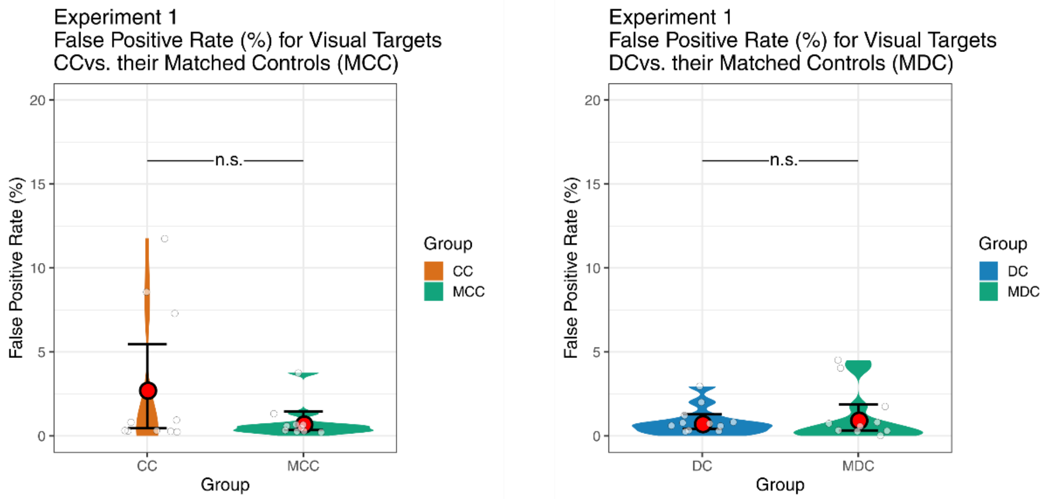

Figure S2. Left: False positive rate (%) for the CC and the MCC group in experiment 1, *N* = 11; Right: False positive rate (%) for the DC and the MDC group in experiment 1, *N* = 13. The red circles indicate group means, with the error bars denoting 95% confidence interval obtained using regular bootstrap with 10,000 replicates [1]. White dots are individual data points jittered for readability.

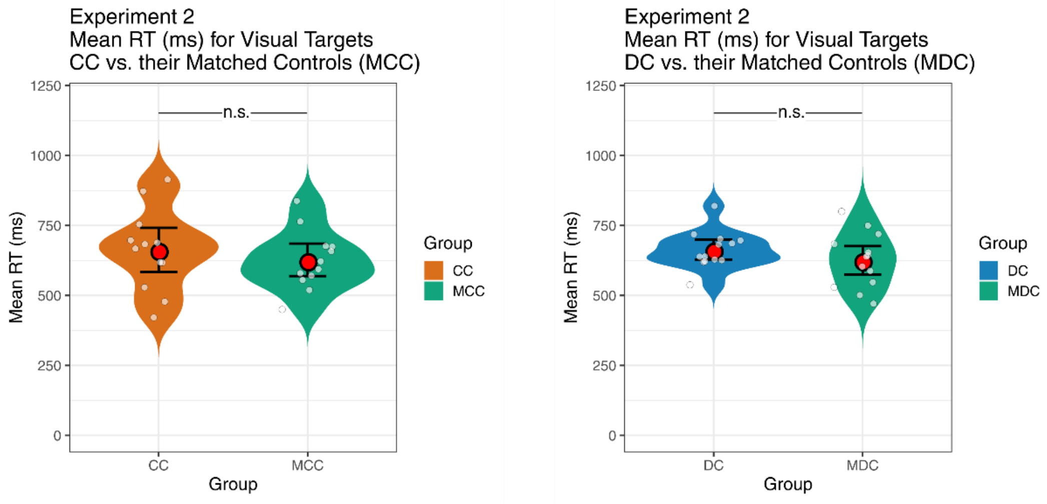

Figure S3. Left: Mean reaction times (RT, ms) for the CC and the MCC group in experiment 2, *N* = 12; Right: Mean RTs (ms) for the DC and the MDC group in experiment 2, *N* = 13. The red circles indicate group means, with the error bars denoting 95% confidence interval obtained using a smoothed bootstrap with a gaussian kernel with 10,000 replicates [1]. White dots are individual data points jittered for readability.

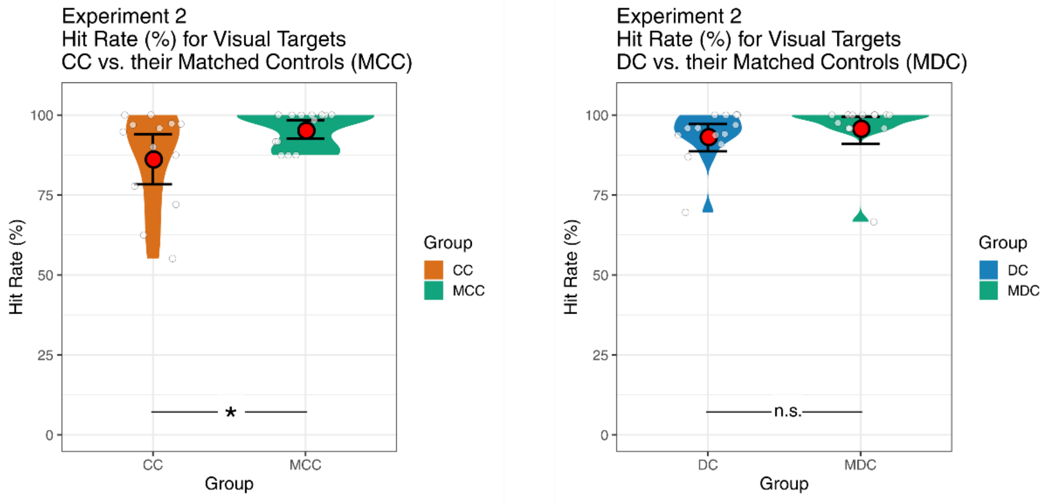

Figure S4. Left: Hit rate (%) for the CC and the MCC group in experiment 2, *N* = 13; Right: Hit rate (%) for the DC and the MDC group in experiment 2, *N* = 13 ($*$: p < .05, one-sided Welch's *t*-test). The red circles indicate group means, with the error bars denoting 95% confidence interval obtained using regular bootstrap with 10,000 replicates [1]. White dots are individual data points jittered for readability.

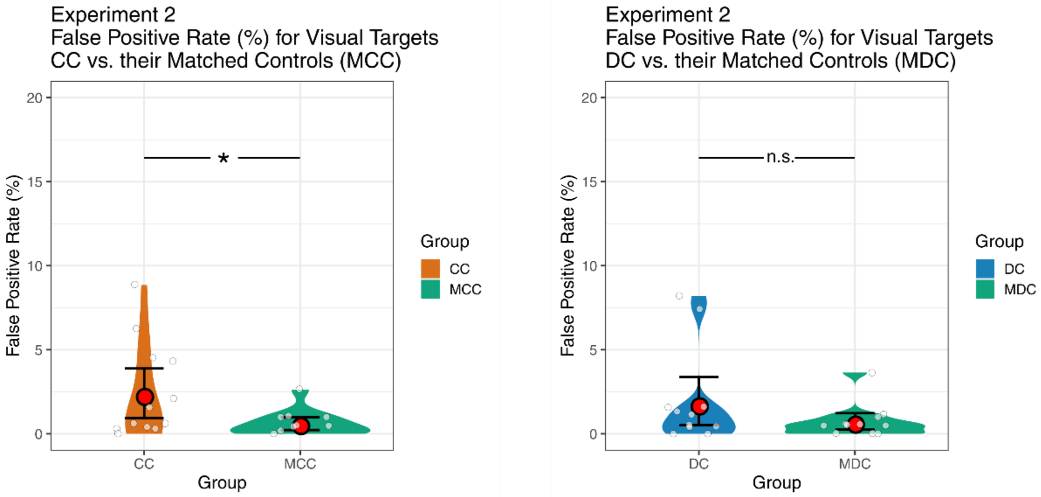

Figure S5. Left: False positive rate (%) for the CC and the MCC group in experiment 2, *N* = 13; Right: False positive rate (%) for the DC and the MDC group in experiment 2, *N* = 13 (∗ : *p* < .05, one-sided Welch's *t*-test). The red circles indicate group means, with the error bars denoting 95% confidence interval obtained using regular bootstrap with 10,000 replicates [1]. White dots are individual data points jittered for readability.

S2.2 Results

In experiment 1, we found that the CC group (*N* = 11) exhibited an average hit rate of 88.6%, *SE* = 3.58%, range = 66.7 – 100%, which was statistically significantly lower than the hit rate of their matched control group (MCC): *N* = 11, *M* = 97.9%, *SE* = 1.86%, range = 79.5 – 100%; one-tailed Welch's *t*-test: *t*(15.022) = −2.330, *p* = .017, Cohen's *h* = −0.402 (see Figure S1). The false positive rate in the CC group, *M* = 2.80%, *SE* = 1.28%, range = 0 – 11.8%, was found not to be statistically significantly different compared to their matched control group (MCC), *M* = 0.786, *SE* = 0.315%, range = 0 – 3.76%, *t*(11.206) = 1.527, *p* = .077 (see Figure S2).

The DC group in experiment 1 (*N* = 13) exhibited an average hit rate of 93.8%, *SE* = 4.14%, range = 45 – 100%, which was not statistically significantly different from the MDC group, *N* = 13, *M* = 96.5%, *SE* = 1.53%, range = 84.2 – 100%; one-tailed Welch's *t*-test: *t*(15.210) = −0.622, *p* = .272 (see Figure S1). Additionally, the false positive rate in the DC group, *N* = 13, *M* = 0.81%, *SE* = 0.23%, range = 0 – 2.94%, was not statistically significantly different from the MDC group, *N* = 13, *M* = 1.01%, *SE* = 0.42%, range = 0 – 4.50%; one-tailed Welch's *t*-test: *t*(18.646) = −0.430, *p* = .664 (see Figure S2).

Analysis of reaction times (RT) in experiment 2 revealed that the CC group (*N* = 12) had an average RT of 662 ms, *SE* = 41.9 ms, range = 421 – 914 ms, which was not statistically significantly different from the RTs of the MCC group, *N* = 12, *M* = 625 ms, *SE* = 30.6 ms, range = 450 – 838 ms, one-tailed Welch's *t*-test, *t*(20.122) = 0.707, *p* = .244 (see Figure S3). Moreover, the CC group in experiment 2 (*N* = 13) exhibited an average hit rate of 86.7%, *SE*

= 4.17%, range = 55.2 – 100%. This was statistically significantly lower than the hit rate of the MCC group: *N* = 13, *M* = 95.7%, *SE* = 1.54%, range = 87.5 – 100%; *t*(15.219) = −2.028, *p* = .030, Cohen's *h* = −0.330 (see Figure S4). The false positive rate in the CC group in experiment 2, *N* = 13, *M* = 2.30%, *SE* = 0.79%, range = 0 – 8.85%, was statistically significantly higher than in the MCC group, *N* = 13, *M* = 0.57%, *SE* = 0.21%, range = 0 – 2.62%; Welch's one-tailed *t*-test: *t*(13.653) = 2.135, *p* = .026, Cohen's *h* = 0.153 (see Figure S5).

The DC group in experiment 2 (*N* = 13) had an average RT of 663 ms, *SE* = 18.6 ms, range = 538 – 820 ms, which was not statistically significantly different from the MDC group, *N* = 13, *M* = 625 ms, *SE* = 27.3 ms, range = 470 – 800 ms; Welch's one-tailed *t*-test: t(21.183) = 1.138, *p* = .134 (see Figure S3). Neither the hit rates, nor the false positive rates statistically significantly differed between the DC and the MDC group; hit rate (DC): *N* = 13, *M* = 93.7%, SE = 2.29%, range = 69.6 – 100%; hit rate (MDC): *N* = 13, *M* = 96.3%, *SE* = 2.52%, range = 66.7 – 100%; one-tailed Welch's *t*-test, *t*(23.776) = −0.767, *p* = .225; false positive rate (DC): *N* = 13, *M* = 1.74%, *SE* = 0.77%, range = 0 – 8.21%; false positive rate (MDC): *N* = 13, *M* = 0.66%, *SE* = 0.272%, range = 0 – 3.65%; one-tailed Welch's *t*-test, *t*(14.985) = 1.335, *p* = .101 (see Figures S4 and S5).

S3. C1 Wave/C1 Effect Based Biomarkers Fail to Differentiate CC Participants

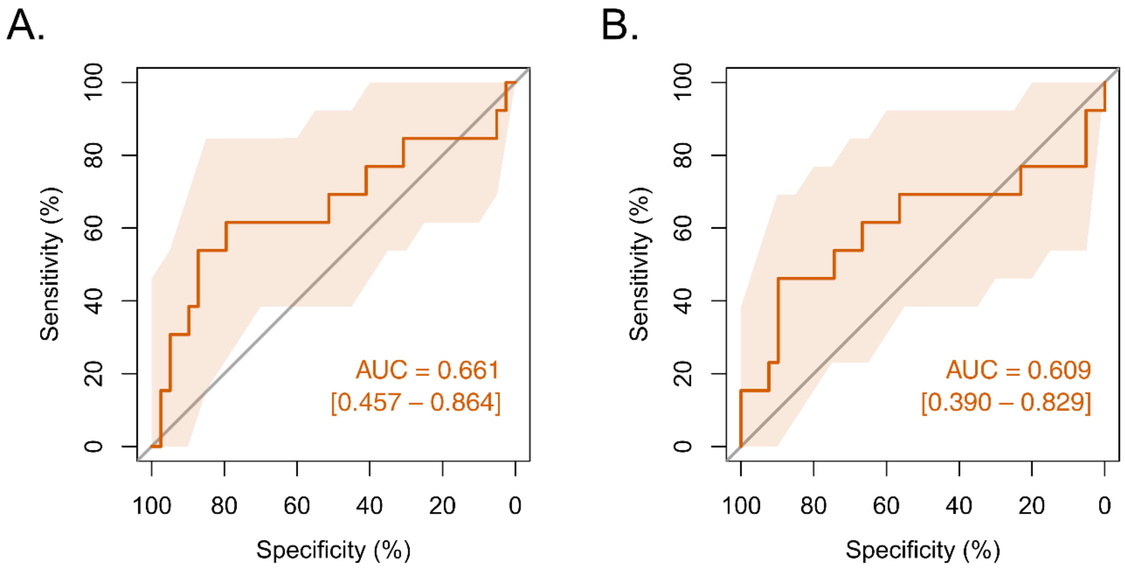

Figure S6. **A.** Biomarker based on normalized C1 wave amplitudes from experiment 1, 50 – 100 ms post-stimulus, using the same methodologies as in the article for the MPP1 biomarker. **B.** Biomarker based on the normalized C1 difference waves from the same experiment [2], that is, the normalized differences between the lower and upper visual field ERPs over posterior electrodes. Confidence intervals (CIs) for the areas under the ROC curves (AUCs) are shown in parentheses and contain the value 0.5. CI bands for sensitivities are depicted as shaded areas (all sensitivity CIs obtained with stratified bootstraps, *n* = 2000).

S4. P1-Based Biomarkers Exhibit High Test-Retest Reliability

Eleven CC, 8 DC, and 5 typically sighted control participants took part in both experiment 1 and experiment 2. For these participants, we calculated two measures of test-retest reliability. First, we calculated Pearson's *r* values for the mean P1 based biomarker (MPP1) and the support vector machine based biomarker (SVMP1) across the two experiments (see Table S5 and Figure S7). More importantly, we calculated Cohen's *κ* as a measure of chance-corrected inter-test classification agreement [3,4]. The classification thresholds were not based on the subset in question but were derived from the complete sample of experiment 1, as in the article (see *Method: Biomarker Development and Statistical Analysis*).

Table S5: Pearson's *r* and Cohen's *κ* as inter-test agreement for the P1-based biomarkers

	**Mean Posterior P1 Biomarker (MPP1)**	**Support Vector Machine Based Biomarker (SVMP1)**
**Pearson's *r***	0.752; $t(22) = 5.345$, $p < .001$	0.833; $t(22) = 7.061$, $p < .001$
**Cohen's *κ***	0.829; z = 4.059, $p < .001$	0.750; $z = 3.687$, $p < .001$

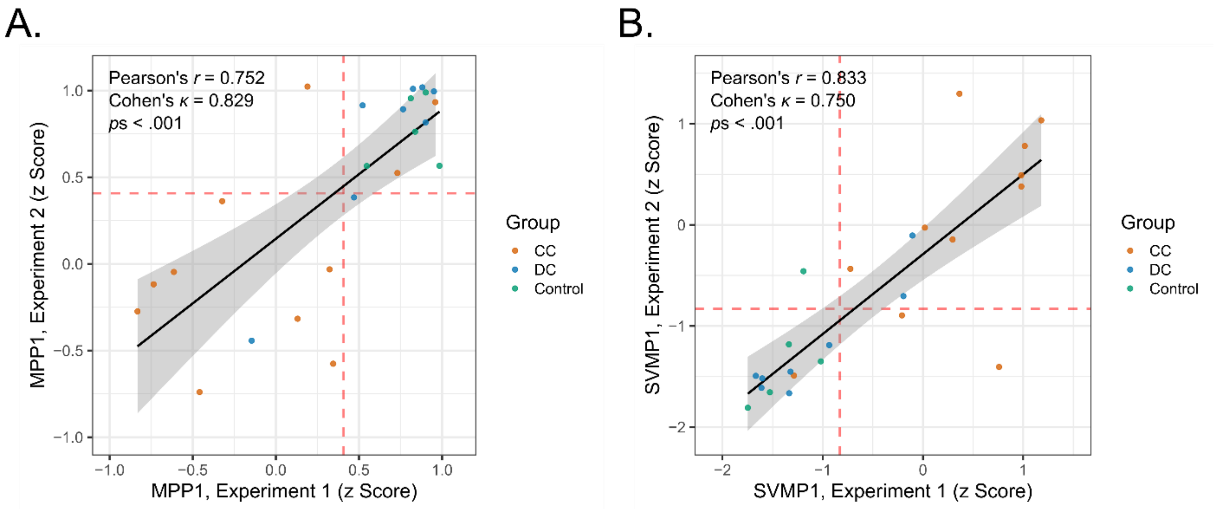

Figure S7. Test-retest reliability for the two electrophysiological biomarkers calculated from the participants who took part in both experiments ($N_{CC} = 11$, $N_{DC} = 8$, $N_{Control} = 5$). **A.** Scatter plot of the mean posterior P1-based biomarker values (MPP1) in the two experiments. CC participants have lower values **B.** Scatter plot of the support vector machine based biomarker values (SVMP1) in the two experiments. CC participants have higher values. Regression line for the best linear fit with error band is shown. Dotted lines indicate the classification threshold value obtained from experiment 1 in the whole group analysis (see *Method: Biomarker Development and Statistical Analysis*).

The high correlation combined with excellent to almost perfect inter-test reliability [3,4] indicates that despite different testing times, viewing distances and response modalities, the

biomarkers exhibited stable performance across participant groups and the two experiments. In the group of 24 participants who took part in both experiments (N_{CC} = 11, N_{DC} = 8, $N_{Control}$ = 5), the inter-test classification agreement for the MPP1 and the SVMP1 biomarker was 92% and 87%, respectively.

References

[1] Wołodźko T. Kernelboot: Smoothed bootstrap and random generation from kernel densities. R package version 0.1.6. CRAN 2019.

[2] Sourav S, Bottari D, Kekunnaya R, Röder B. Evidence of a retinotopic organization of early visual cortex but impaired extrastriate processing in sight recovery individuals. J Vis 2018;18:22. doi: 10.1167/18.3.22.

[3] Cicchetti DV, Rourke BP. Reliability. In: Methodological and biostatistical foundations of clinical neuropsychology and medical and health disciplines. 2nd ed., Lisse: Psychology Press; 2004, p. 17–23.

[4] Landis JR, Koch GG. The Measurement of Observer Agreement for Categorical Data. Biometrics 1977;33:159–74. doi: 10.2307/2529310.
