# Supplementary material for: An electrophysiological biomarker for the classification of cataract-reversal patients: A case-control study
Source: eClinicalMedicine. 2020 Oct 6;27:100559. doi: 10.1016/j.eclinm.2020.100559 (PMC7548424; doi:10.1016/j.eclinm.2020.100559)
Supplement: Supplementary file 3 [file mmc3.pdf]

Consent for Acknowledgements

I, the undersigned D. BALASUBRAMANIAN agree to be acknowledged in the work entitled *An Electrophysiological Biomarker for the Classification of Cataract-Reversal Patients*, currently in consideration for publication in the journal EClinicalMedicine (Manuscript ID: eclinm-D-20-00043)

Signed on 13/05/2020, at (place) Hyderabad, India

Name: D. Balasubramanian

Signature: [illegible]

Consent for Acknowledgements

I, the undersigned Seema Banerjee

agree to be acknowledged in the work entitled *An Electrophysiological Biomarker for the Classification of Cataract-Reversal Patients*, currently in consideration for publication in the journal EClinicalMedicine (Manuscript ID: eclinm-D-20-00043)

Signed on 17 / 05 / 2020, at (place) Hong Kong

Name: Seema Banerjee

Signature: Banerjee

Consent for Acknowledgements

I, the undersigned Larissa Brockmann

agree to be acknowledged in the work entitled *An Electrophysiological Biomarker for the Classification of Cataract-Reversal Patients*, currently in consideration for publication in the journal EClinicalMedicine (Manuscript ID: eclinm-D-20-00043)

Signed on 15 / 05 / 2020, at (place) Hamburg

Name: Larissa Brockmann

Signature: L. Brockmann

Consent for Acknowledgements

I, the undersigned MARIA GUERREIRO

agree to be acknowledged in the work entitled *An Electrophysiological Biomarker for the Classification of Cataract-Reversal Patients*, currently in consideration for publication in the journal EClinicalMedicine (Manuscript ID: eclinm-D-20-00043)

Signed on 13 / 05 / 2020, at (place) HAMBURG (GERMANY)

Name: MARIA GUERREIRO

Signature: Maria Guerreiro.

Consent for Acknowledgements

I, the undersigned Kabilan Pitchaimuthu

agree to be acknowledged in the work entitled *An Electrophysiological Biomarker for the Classification of Cataract-Reversal Patients*, currently in consideration for publication in the journal EClinicalMedicine (Manuscript ID: eclinm-D-20-00043)

Signed on 13 / 05 / 2020, at (place) Chennai, India

Name: Kabilan Pitchaimuthu

Signature: ____________________

Consent for Acknowledgements

I, the undersigned Siddhart Srivatsav Rajendran

agree to be acknowledged in the work entitled *An Electrophysiological Biomarker for the Classification of Cataract-Reversal Patients*, currently in consideration for publication in the journal EClinicalMedicine (Manuscript ID: eclinm-D-20-00043)

Signed on 16 / 5 / 2020, at (place) Hyderabad, Telangana, India

Name: Siddhart Srivatsav Rajendran

Signature: X Signed electronically

Consent for Acknowledgements

I, the undersigned Lisa Stockleben agree to be acknowledged in the work entitled *An Electrophysiological Biomarker for the Classification of Cataract-Reversal Patients*, currently in consideration for publication in the journal EClinicalMedicine (Manuscript ID: eclinm-D-20-00043)

Signed on 15 / 05 / 2020, at (place) Cologne, Germany

Name: Lisa Stockleben

Signature: X lisa.stockleben@uni-koeln.de

Consent for Acknowledgements

I, the undersigned FLORIAN SÜßER

agree to be acknowledged in the work entitled *An Electrophysiological Biomarker for the Classification of Cataract-Reversal Patients*, currently in consideration for publication in the journal EClinicalMedicine (Manuscript ID: eclinm-D-20-00043)

Signed on 13 / 05 / 2020, at (place) Hamburg, GERMANY

Name: FLORIAN SÜßER

Signature: [illegible]
